# Evaluating the effects of red imported fire ants (*Solenopsis invicta*) on juvenile Houston Toads (*Bufo* [=*Anaxyrus*] *houstonensis*) in Colorado County, TX

**DOI:** 10.7717/peerj.8480

**Published:** 2020-02-10

**Authors:** Shashwat Sirsi, Madeleine J. Marsh, Michael R.J. Forstner

**Affiliations:** Department of Biology, Texas State University, San Marcos, TX, United States of America

**Keywords:** Attwater Prairie Chicken National Wildlife Refuge, Endangered Species, Invasive Species, Experimental Study, Growth, Survival, Recovery Efforts

## Abstract

The spread of invasive species is considered a major threat to biodiversity, second only to habitat loss. Red Imported Fire Ants (*Solenopsis invicta*) are a globally invasive species with negative impacts reported on native invertebrate and vertebrate species. Federally endangered Houston Toads (*Bufo* [=*Anaxyrus*]* houstonensis*), endemic to Texas, are among the vertebrates reportedly negatively impacted by Red Imported Fire Ants (RIFA). Threats posed by RIFA to Houston Toads needed to be explicitly characterized. Large-scale chemical treatments to suppress RIFA and facilitate brood survival in Attwater’s prairie-chickens (*Tympanuchus cupido attwateri*) at the Attwater Prairie Chicken National Wildlife Refuge (APCNWR) afforded us an opportunity to experimentally examine the influence of RIFA abundance on juvenile Houston Toad growth and survival. We also sought to examine whether juvenile Houston Toads could grow and survive in a vegetation type similar to a historic species locality. We conducted a terrestrial mesocosm experiment to test whether the application of bait-driven suppressant decreased counts of RIFA relative to untreated sites. We examined whether counts of native ant and non-ant native invertebrates were higher in response to potential decreases in RIFA. We compared growth and survival rates in juvenile Houston Toads among treated and untreated sites, expecting juvenile growth and survival to be higher in response to potentially decreased RIFA counts and increased native invertebrate counts. We saw lower counts of RIFA in treated prairies, but we also observed a decrease in native ant counts possibly due to chemical treatment. Therefore, the application of bait-driven suppressant may not affect RIFA alone. We saw no difference in counts of non-ant invertebrates among treated and untreated sites. Juvenile Houston Toads did not differ in growth and survival among treated and untreated sites. We recognize that the lack of a relationship between juvenile growth and survival with a treatment effect, and therefore RIFA abundance, may be limited to APCNWR. We encourage additional experimental studies to elucidate RIFA impacts at other sites. We extrapolated apparent survival estimates from our study to one year. These appear comparable to juvenile survivorship required in simulations for Houston Toad population persistence and on this basis, we recommend that APCNWR be re-evaluated as a reintroduction site for Houston Toads. We also recommend further studies to potentially broaden the regulatory definition of Houston Toad habitat beyond the current restrictive view of canopied forest alone. Such studies would need to examine the utility of native grasslands as dispersal corridors/upland habitat for juvenile Houston Toads. Our findings emphasize the utility of experimental studies in directly examining the influence of perceived threats to imperiled species and the role of such clarifications in adapting recovery efforts.

## Introduction

The introduction and spread of non-native species is considered a major threat to biodiversity, second only to habitat loss as a cause of species endangerment and extinction (Wilcove et al., 1998; Mooney & Cleland, 2001; Gurevitch & Padilla, 2004; Clavero & Garcia-Berthou, 2005; Pimentel, Zuniga & Morrison, 2005). In the United States, Wilcove et al. (1998) quantified data on threats to imperiled species and reported that ∼50% of native species were threatened by alien-invasive species. A lack of effective predators and land-use alterations that result in favorable habitat potentially facilitate high abundances of several of the estimated 50,000 alien-invasive species in the United States (Pimentel, Zuniga & Morrison, 2005). The economic consequences of damage caused by invasive species are considerable with estimates ranging from $1.1 billion/year (OTA, 1993) to $120 billion/year (Pimentel, Zuniga & Morrison, 2005).

Red Imported Fire Ants (*Solenopsis invicta*), hereafter RIFA, are iconic among invasive species in the United States. RIFA were accidentally introduced to the United States near Mobile, Alabama ca. 80 years ago (Porter & Savignano, 1990; Wilcove et al., 1998). The species subsequently rapidly spread and is now established in southern and southeastern US (Porter & Savignano, 1990; Ascunce et al., 2011). At high densities, RIFA are reported to negatively impact species diversity and abundance of native ants and other surface-active arthropods (Porter & Savignano, 1990). Studies have also implicated RIFA as causal factors in vertebrate range extirpations, population declines, and negatively influencing juvenile recruitment (Landers, Garner & McRae, 1980; Mount, Trauth & Mason, 1981; Price, 1990; Goin, 1992; Allen, Lutz & Demarais, 1995; Allen et al., 2001; Buhlmann & Coffman, 2001; Parris, Lamont & Carthy, 2002; Epperson & Heise, 2003). RIFA-associated damage to wildlife, livestock, and public health in Texas alone is estimated at $300 million/year (Pimentel, Zuniga & Morrison, 2005). However, Gurevitch & Padilla (2004) contend that while population declines and range contractions in native species may be predominantly attributed to the spread of alien-invasive species, they may also be correlated with another threat such as habitat alteration. For instance, declines in northern bobwhite (*Colinus virginianus*) populations have been a well-known cause of concern over the past two decades (Hernández et al., 2013). These population declines have been attributed to land-use change (Brennan, 1991; Williams et al., 2004) and predation by RIFA (Allen, Lutz & Demarais, 1993; Allen, Lutz & Demarais, 1995). Hernández et al. (2013) note that northern bobwhites may suffer increased RIFA predation rates in fragmented habitat. The causes of decline may thus be conflated. Such confounding factors necessitate experimental or quantitative studies to specifically determine the role of RIFA in population declines and/or range contractions of native species. Such studies would then help adapt recovery efforts for imperiled species.

Houston Toads (*Bufo* [=*Anaxyrus*] *houstonensis*) are among the imperiled native species that may be negatively affected by RIFA. Houston Toads have been in continuous decline since their first description in 1953, primarily due to habitat loss and degradation (Sanders, 1953; Brown, 1975; Brown & Mesrobian, 2005). These amphibians have a restricted range with a currently known distribution in nine Texas counties (i.e., Austin, Bastrop, Burleson, Colorado, Lavaca, Lee, Leon, Milam, and Robertson; USFWS, 2011; Fig. S1). RIFA occur throughout this known range and are considered a major threat as they may predate emergent juvenile and adult toads as well as deplete the species’ arthropod prey base (Bragg, 1960; Clarke, 1974; Potter Jr et al., 1984; Freed & Neitman, 1988; Porter & Savignano, 1990; IUCN/SSC Conservation Breeding Specialist Group, 1994). Owing to these potential impacts, the presence of RIFA at the Attwater Prairie Chicken National Wildlife Refuge is considered to have hindered an attempted reintroduction of Houston Toads at this site (Quinn & Mays, 1987; Dodd & Seigel, 1991; IUCN/SSC Conservation Breeding Specialist Group, 1994; Forstner & Dixon, 2010).

The impact of RIFA on amphibians has been examined in previous published studies, but these are few relative to studies that document RIFA interacting with other taxa (Todd et al., 2008; Long et al., 2015). Additionally, aside from a single event reporting multiple observations of RIFA predation on emergent juvenile *B. houstonensis* and a subsequent study that correlated peak RIFA activity and juvenile emergence (Freed & Neitman, 1988; Brown, DeVolld & Forstner, 2012), no published account has explicitly characterized the threats that RIFA may pose to the persistence of Houston Toad populations. We attempted to address this gap by applying an experimental approach to evaluate the effect of RIFA presence on growth and survival of juvenile Houston Toads.

We conducted this study at the APCNWR, as this prairie was within the historic range of the Houston Toads and subject to landscape level chemical treatment for RIFA to promote brood survival in Attwater’s prairie-chickens (*Tympanuchus cupido attwateri*, (Caldwell, 2015; Morrow et al., 2015). This enabled our primary goal of comparing growth and survival in juvenile Houston Toads among sites that potentially had relatively high (i.e., untreated) and low (i.e., treated) RIFA abundance. We expected that a higher number of RIFA at untreated sites would have a detrimental effect on both the growth and survival of juvenile Houston Toads due to diminished abundance of native invertebrates (i.e., exploitative competition by RIFA) as well as due to RIFA predation on juvenile toads. For this purpose, our study sought to compare counts of RIFA, counts of native ants and non-ant invertebrates, and growth and survival in Houston Toads among treated and untreated sites. Additionally, while Houston Toads were historically documented from locations in coastal prairies with deep sandy soils (Kennedy, 1962; Potter Jr et al., 1984), the relatively modern paradigm primarily associates Houston Toad habitat with forested areas that have sandy soils (Buzo, 2008; Forstner & Dixon, 2010). The intended study at APCNWR thereby also enabled a secondary goal of observing whether juvenile Houston Toads could grow and survive in a habitat type that has historic connotations but is currently not included in the restrictive definition of Houston Toad habitat.

## Materials & Methods

### Study site

**Figure 1:**
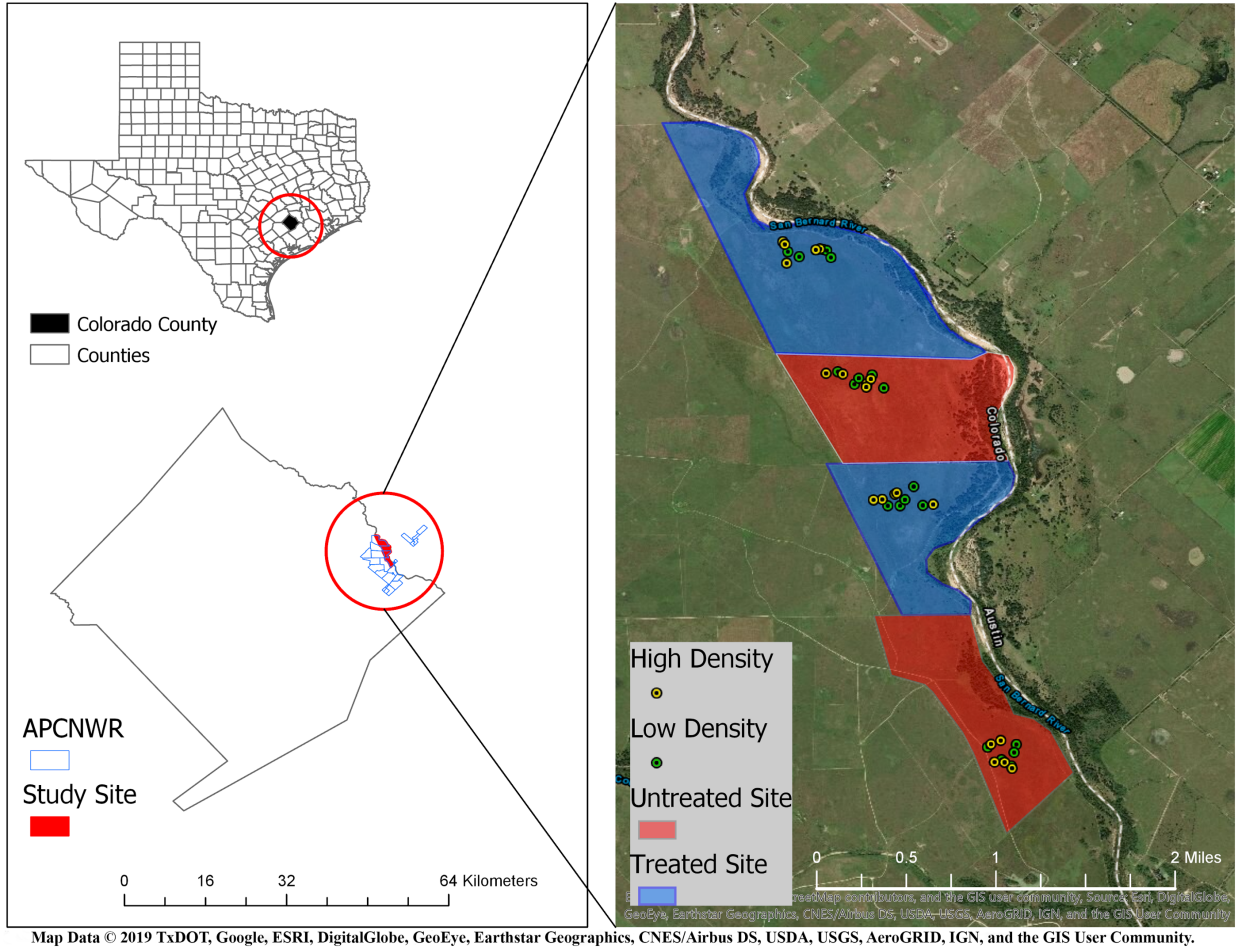
Location of study area in Attwater Prairie Chicken National Wildlife Refuge (APCNWR), and its geographical location within Colorado County, TX. Four prairies were included in the study conducted from March–September 2015, two of which (blue areas) were treated for Red Imported Fire Ants (RIFA) aerially with insecticidal bait while the other two (red areas) were left untreated. 10 experimental units or exclosures were assigned locations via random generation of spatial coordinates within each prairie and contained either four (green dots) or six (yellow dots) juvenile Houston Toads (*Bufo* [=*Anaxyrus*] *houstonensis*) per exclosure. Map Data ©2019 Texas Department of Transportation, Google, ESRI, DigitalGlobe, GeoEye, Earthstar Geographics, CNES/Airbus DS, USDA, USGS, AeroGRID, IGN, and GIS User Community.

We conducted this study on the 4,265 ha APCNWR (Colorado County, Texas) which is about 120 km inland from the Gulf of Mexico (Morrow et al., 1996). The refuge is predominantly prairie grasslands with loamy prairie, sandy prairie, and coarse sand range sites (Morrow et al., 1996). This site is a unit of the USFWS National Wildlife Refuge System and represents one of the largest remaining patches of coastal prairie habitat. It is managed for the federally endangered Attwater’s prairie-chicken *(Tympanuchus cupido attwateri)* through prescribed fires, cattle grazing, native seeding, and RIFA control. The climate in these coastal prairies is subtropical with average annual precipitation ranging from approximately 750 mm to 1,100 mm (Kwiatkowski, Saenz & Hibbitts, 2017). The San Bernard River borders APCNWR to the east. For our field experiment, we selected four prairies (Fig. 1) within APCNWR, each of which were coarse sand range sites to enable burrowing by Houston Toads (Caldwell, 2015). These open grasslands were dominated by mid-tall native grass species (Morrow et al., 2015).

### Experimental design

We conducted this study over a total area of 739.5 ha. Of that total, 261.5 ha were left untreated and 478 ha were treated for RIFA using 1.7 kg/ha of Extinguish^^®^^ Plus (0.25% s-methoprene, 0.36% hydramethylnon; Central Life Sciences, Schaumburg, IL) on 22 October 2014 (Fig. 1). An insecticide and insect growth regulator in this bait-driven suppressant is reported to disproportionately affect RIFA in comparison to native ant species (Calixto et al., 2007). A helicopter was used to place aerial applications of Extinguish^^®^^ Plus on two (i.e., treated zones) of the four prairies selected for our study (Fig. 1). We adhered to manufacturer’s protocol with regard to the application rate of the insecticidal bait (Wellmark^^®^^/Central Life Sciences of Central Garden & Pet). RIFA from small colonies have been observed to forage no further than 3 m for food while those from large colonies showed foraging distances of up to 17 m (Stringer et al., 2011). To account for the upper-bound of foraging distances and ensure no overlap between treatments, we created a 50-m buffer from the edge of each treatment. Further, we also restricted the random assignment of experimental unit locations within 250 × 500 m plots that were approximately within the center of each of our four prairies. This sought to control for any overspray of insecticidal bait from treated into untreated prairies, or any adjacency effects of the treatments themselves.

### Assessing RIFA and native invertebrate density

We assessed RIFA densities among treated and untreated prairies starting on 8 April 2015, every two weeks through July 2015. We evaluated RIFA activity using 6 bait traps per prairie. Each bait trap consisted of pureed, canned sausages in a petri-dish and we placed these bait traps at randomly assigned locations between access roads and the edge of our treatment sites. Bait traps in each prairie were placed at least ∼10 m apart from each other. Given that these were baits of meat and likely to be monopolized by a dominant, predacious species (Greenslade & Greenslade, 1971), we restricted trap locations, as above, so as to not directly attract RIFA to our outdoor experimental units (i.e., toad exclosures). Bait traps were set for a period of ∼3 h (∼10:00AM–1:00PM). We concurrently sampled for surface-active native invertebrates using wet pitfall traps. These were High Density Polyethylene cups of ≈120 ml volume that contained propylene glycol (commercial non-toxic antifreeze). Pitfall traps were placed 15 m north of toad exclosures in each prairie, to prevent attracting ants to those exclosures. Pitfall traps were open for a duration of 24 h, though not on the same day as bait traps. We expected pitfall traps were less likely to be dominated by predacious species because this is a more passive sampling technique (Greenslade & Greenslade, 1971). We identified native ants to the genus level and classified non-ant native invertebrates as small (1 to 12 mm body length) or large (>12 mm body length). We counted the total number of RIFA, native ants, and non-ant native invertebrates in each pitfall trap. These pitfall traps were primarily used to quantify the number of available prey items (i.e., ground-dwelling/surface active native invertebrates) for Houston Toads with respect to RIFA treatment. Locations for bait and pitfall traps were marked by surveyor flags and recorded in a handheld GPS unit to enable repeated visits.

We pooled observations for RIFA, native ants, and non-ant native invertebrates by treated and untreated sites, from bait and pitfall traps. The number of observations from pitfall and bait traps (*n* = 194) were an order of magnitude lower than repeated measures of individual toads (*n* = 1,339). This difference was because our sampling intensity for toads was higher than invertebrates and did not allow us to use counts of RIFA directly as a predictor of growth and survival in juvenile toads. Such a direct comparison would lose the variation inherent in our toad dataset by pooling repeat measurements or erroneously pseudoreplicate our RIFA counts. For this reason, we employed a syllogistic approach of first testing for differences in counts of RIFA and native ants and non-ant invertebrates among prairies that were treated and untreated for RIFA and then testing for a treatment effect in toad growth and survival.

All analyses were performed using Program *R* (R Core Team, 2016). Where relevant, we inferred significance at *α* = 0.05. Mean values are reported with standard error. We fitted separate GLMMs, using Poisson and Negative Binomial response distributions with and without zero-inflation, to our RIFA, native ant, and non-ant native arthropod counts in the *R* package *glmmADMB* (Fournier et al., 2012). We attempted to account for repeated measures of our prairies by including individual prairies as a random factor. Candidate models included random variation in intercepts and random variation in slopes and intercepts. We evaluated model fit using AIC_*c*_ values in package *MuMIn* (Barton, 2017). Models with a Δ*AIC*_*c*_ ≤ 2 were considered similar to models that best supported the data. Once we had chosen optimal random structure, we assessed error distributions and evaluated our fixed factors of treatment and time (i.e., week). A subset of candidate models (Tables S1–S3) specified an interaction among time (i.e., week) and treatment to account for continued ant suppression over time and the expected benefits of such RIFA decreases on native invertebrate assemblages (Calixto et al., 2007). The fixed effect structure that best supported the data was chosen using AIC_*c*_ values as above.

### Juvenile Houston toad growth and survivorship

We treated 478 ha of our study area for RIFA using 1.7 kg/ha of Extinguish^^®^^ Plus (0.25% s-methoprene, 0.36% hydramethylnon; Central Life Sciences, Schaumburg, IL) on 22 October 2014. We began our study in March 2015, which was within the estimated 12-month duration of RIFA control by insecticidal bait (Wellmark^^®^^/Central Life Sciences of Central Garden & Pet). We used outdoor terrestrial experimental units (Hopkins, Mendonça & Congdon, 1997) to track growth and survival in juvenile Houston Toads at our treatment sites. We assigned locations for ten experimental units in a 250 × 500 m area of each prairie via random generation of spatial coordinates, for a total of 40 experimental units (Fig. 1).

**Figure 2:**
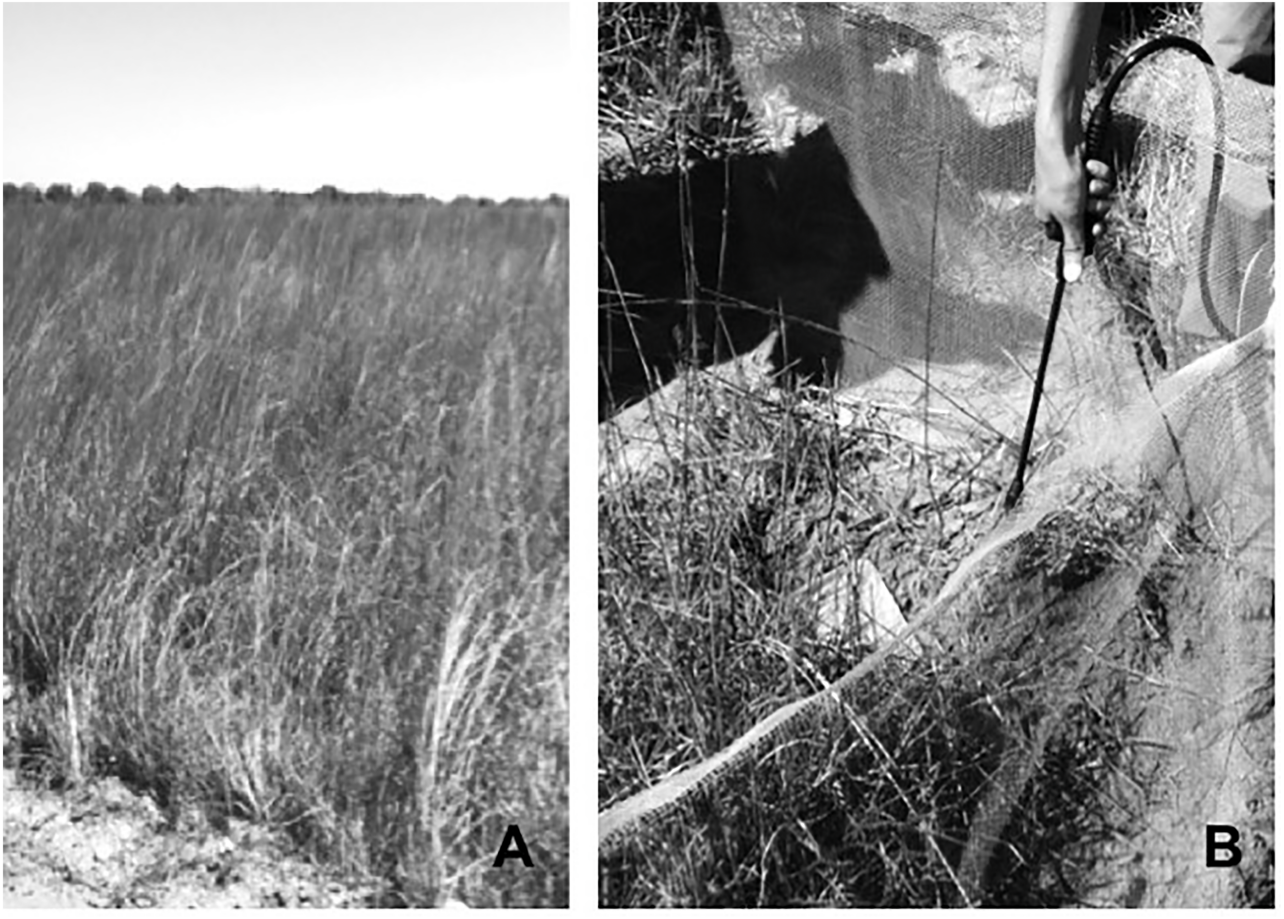
Open grassland habitat at Attwater Prairie Chicken National Wildlife Refuge (A) where exclosures (B) were deployed to test the effect of invasive Red Imported Fire Ants on juvenile Houston Toads. We set up 40 experimental units/exclosures at APCNWR to track growth and survival of Houston Toads in coastal prairie vegetation (A) from March–September 2015. Each experimental unit (B) or exclosure enabled juvenile toad survival while excluding potential vertebrate predators. Each exclosure was a 150 × 150 cm area of the ground enclosed by hardware cloth (3.2 mm mesh size, 0.36 mm wire diameter). We moistened small plastic tubs full of organic compost and sphagnum moss within exclosures, whenever necessary to mitigate chances of toad desiccation. Photo Credit: Madeleine J. Marsh.

Each experimental unit, hereafter exclosure, was a 150 × 150 cm area of the ground enclosed by hardware cloth (3.2 mm mesh size, 0.36 mm wire diameter; Fig. 2). We refer to our experimental units as exclosures for their ability to exclude potential vertebrate predators (e.g., snakes, birds). Exclosure walls extended 20 cm below ground and 50 cm above ground. We folded a 10 cm lip of hardware cloth along the length of the top and bottom of each exclosure to prevent toads from escaping by tunneling under or scaling exclosure walls to climb out (Jones, 2015). Additionally, we placed deer netting on top of each exclosure to prevent entry of predators. However, the hardware cloth we used had a mesh size (3.2 mm) that was large enough to enable RIFA to move in and out of exclosures. We mitigated chances of desiccation of individuals by placing two small plastic water tubs full of organic compost and sphagnum moss within the artificial containment. We placed each tub under or in the shelter of the native bunch grasses within each of the exclosures. We recorded exclosure locations in handheld GPS units, which enabled repeated visits to the exclosures.

We randomly assigned and released 200 full sibling, juvenile toads in exclosures on 25 March 2015. These juveniles were sourced from the captive breeding program at Houston Zoo. We aimed to minimize heterogeneity by using full sibling toads (i.e., from the same clutch). Houston Toads were used immediately after metamorphosis was complete (defined as total absorption of tail) and did not appear to show considerable difference in size among exclosures in treated (Body Mass (W in g) = 0.79 ±  0.03 g; Snout Urostyle Length (SUL in mm) = 17.89 ± 0.26 mm; Mean ± SE) and untreated sites (*W* = 0.75 ± 0.04 g; SUL = 18.2 ± 0.35 mm; Mean ± SE). Juvenile release densities in each exclosure were based off a previous study conducted in canopied habitat in Bastrop County, Texas (Jones, 2015). Jones (2015) observed that survivorship in Houston Toads was best explained by release density, with a release density of five (*ϕ* = 0.93) toads per exclosure having the highest survivorship followed closely by exclosures that had six (*ϕ* = 0.92) and four (*ϕ* = 0.91) toads. Recapture rates in exclosures that had four (*p* = 0.91) and six (*p* = 0.92) toads were higher than those exclosures which had five (*p* = 0.87) toads (Jones, 2015). We released four juveniles per exclosure in 5 exclosures per prairie (*n* = 20 exclosures) and six juveniles per exclosure in the remaining 5 exclosures per prairie (*n* = 20 exclosures). We varied juvenile densities in exclosures as above because these densities potentially ensured study longevity via a trade-off between survival and recapture rates (Jones, 2015) and the number of available captive toads. Secondarily, variation in release density also allowed us to extend a test of these previously determined optimal release densities (Jones, 2015) in coastal prairie habitat. We marked each individual by clipping toes using sterile dissection scissors in correspondence to a specific numbering system (i.e., number 0, 1, 2, 3 for 4 juveniles per exclosure and 0, 1, 2, 3, 4, 5 for 6 juveniles per exclosure; (Ferner, 1979). We weighed and measured juveniles prior to placing them in exclosures. Marked individuals allowed for tracking of individual growth and survival. Our study was authorized by US Fish and Wildlife Service (Permit No. TE039544-0) and Texas Parks and Wildlife Department (Permit No. SPR-0102-191). Our study protocol was approved by the Texas State University Institutional Animal Care and Use Committee (Protocol No. IACUC201648186).

Previous studies report reduced survival in translocated amphibians immediately following release (Cooke & Oldham, 1995; Tocher & Pledger, 2005). For this reason, we conducted daily searches of exclosures for toads over five days immediately following release and sprayed water tubs to keep them moist. Following the initial daily searches, data were collected from the individuals for approximately six months; we recorded W in g, SUL in mm, and head width (HW in mm) for each individual recaptured after searching the exclosure for five minutes. We searched exclosures and measured toads weekly from Week 2 to 6. Subsequently we searched exclosures every two weeks from Week 8 to 16. We conducted the last two exclosure searches on Week 20 and 24. Toad desiccation would likely have been exacerbated by artificial containment and so we mitigated this by moistening water tubs in the exclosures after five days of no precipitation. We sampled more frequently at the start of the study in order to monitor for low survival following release and to account for an inherently low survival rate in Houston Toads of a smaller size class (Cooke & Oldham, 1995; Tocher & Pledger, 2005; Greuter, 2004; Swannack, Grant & Forstner, 2009). As Houston Toads increased in size and thereby survivorship, we decreased sampling frequency to reduce the level of disturbance (Greuter, 2004; Swannack, Grant & Forstner, 2009). Exclosures were searched by multiple observers, including Texas State University students, Student Conservation Association (SCA) interns, and APCNWR staff members. In order to specifically control for observer bias in measuring toads, all observers were trained prior to their participation in the study. In addition, the role of taking measurements was restricted to the same subset of observers over repeat occasions.

Juvenile body mass is likely to vary as a function of both growth and hydration status (Todd & Rothermel, 2006). We therefore used SUL in mm as a more stable proxy for growth. We used linear mixed effects models in package *lme4* (Bates et al., 2015) to address the primary question on whether juvenile growth varied among treated and untreated sites, while attempting to account for other sources of variation. We used the beyond optimal approach as described by Zuur et al. (2009), i.e., we included as many explanatory variables in the fixed component of the model- so that the random component did not have any information that we would instead need in the fixed component of the model. We fitted models with all explanatory variables and as many interactions as possible, i.e., a three-way interaction of fixed factors of treatment, time (i.e., week), and release density while varying random factors to determine optimal random effect structure. Models were fit using Restricted Maximum Likelihood (REML) estimation and optimal structure of random factors was assessed using Akaike Information Criterion scores corrected for a small sample size (AIC_*c*_) in package *MuMIn* (Barton, 2017). Models with a ΔAIC_*c*_ ≤ 2 were considered similar to models that best supported the data. Once the structure of our random factor was chosen, we fitted models with varying fixed factor structure using Maximum Likelihood (ML) estimation and assessed fixed factor structure using AIC_*c*_ values as above. We then presented our best-fit model using REML estimation, where *t* tests on such estimates used Satterthwaite approximations to degrees of freedom (Zuur et al., 2009).

We accounted for non-independence of repeated measures of toads in exclosures by testing each individual toad, each exclosure, and each individual toad within an exclosure as a random factor. Candidate models included random variation in intercepts and random variation in intercepts and slopes (Table S4). We tested for potential differences in SUL among fixed factors of treatment (i.e., treated or untreated), initial/release density (i.e., four or six juveniles), and time (i.e., week), as well as higher order interactions of these fixed factors (Table S4).

We estimated juvenile survivorship by applying a Cormack–Jolly–Seber model to live recapture data in Program *MARK* (Cormack, 1964; Jolly, 1965; Seber, 1965; White & Burnham, 1999). We used the *RMark* package to interface with Program *MARK* from the *R* environment (Laake, 2013; Mazerolle, 2015). Program *MARK* employs information-theoretic methods in selecting the best supported model estimates of apparent survival (*ϕ*) and recapture probability (*p*). We explored variation in *ϕ* and *p* using time and treatment (i.e., treated and untreated sites) as well as higher-order interactions of time and treatment as explanatory variables. Additionally, we also included models that considered either or both parameters as constant among sampling periods. We developed 14 candidate models (Table S5), with each model representing a distinct biologically-relevant hypothesis. We estimated the overdispersion parameter ($\hat {c}$) by dividing the global *χ*^2^ by its corresponding degrees of freedom (Mazerolle, 2015). The overdispersion parameter was incorporated to provide Quasi-likelihood Akaike Information Criterion scores corrected for a small sample size (QAIC_*c*_), based off which model fit was evaluated. Models with a ΔQAIC_*c*_ ≤ 2 were considered similar to models that best supported the data; however, we report models over a cumulative weight of ≈0.95.

## Results

### Assessing RIFA and native invertebrate density

**Figure 3:**
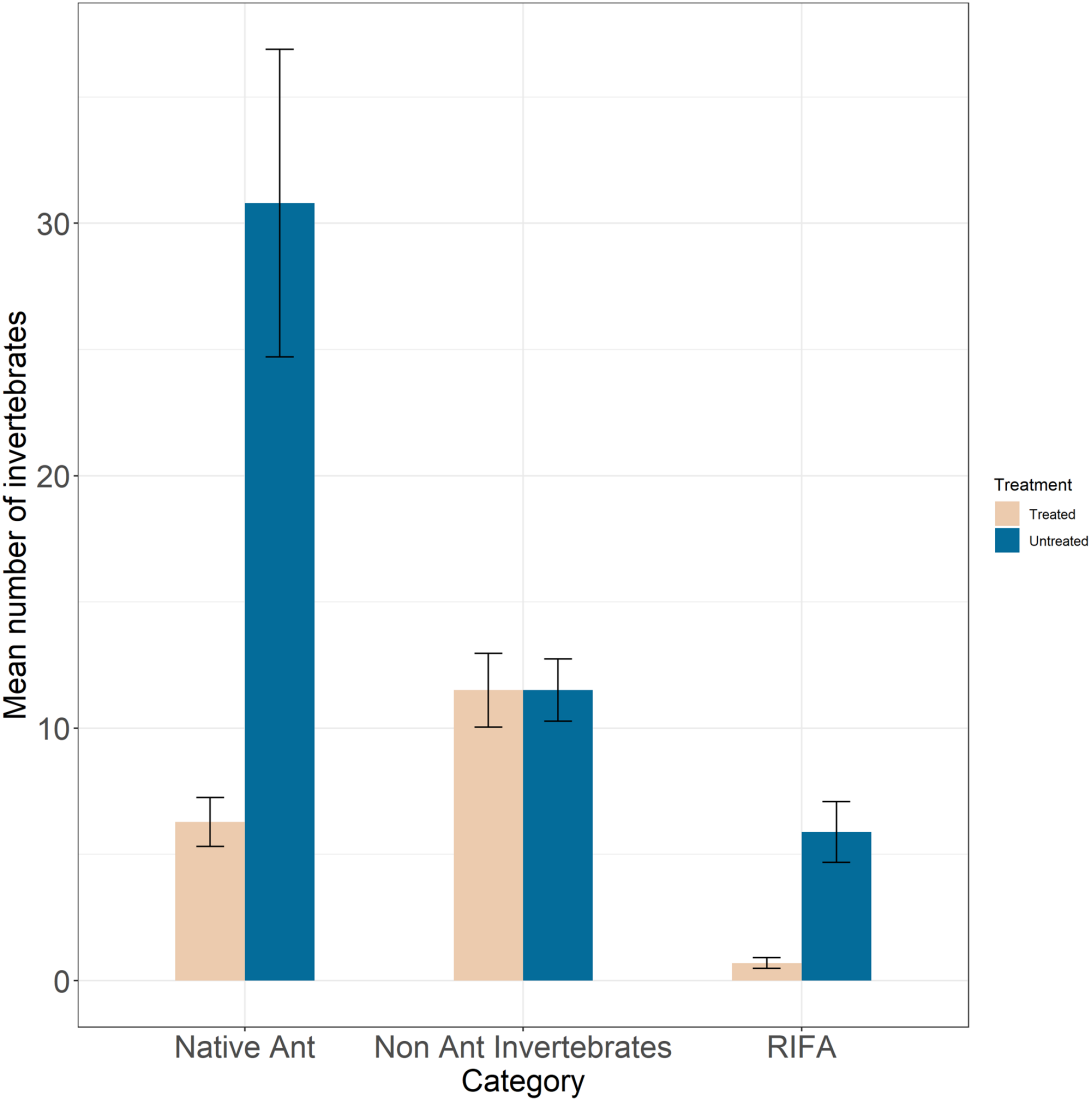
Mean number of invertebrates (*y*-axis) categorized by Red Imported Fire Ants (RIFA), native ants, and non-ant native invertebrates (*x*-axis) in treated (tan bars) and untreated (blue bars) areas. Mean counts of RIFA in untreated areas were nearly nine times greater than RIFA counts in treated prairies. Mean counts of native ant species were nearly five times greater than native ant counts in treated areas. The mean number of non-ant native invertebrates were nearly equal among treated and untreated areas.

The mean number of RIFA per sample in untreated sites (5.89 ± 1.2) were nearly 9 times greater than that in treated sites (0.695 ± 0.21 ants; Fig. 3). Our best-fit model (Table S1) was one that fit RIFA counts to a type-two negative binomial distribution (i.e., quadratic mean–variance relationship) with intercepts randomly varying among prairies. This model showed a greater rate of decrease in the number of RIFA in treated areas relative to untreated areas (*β* = −0.257, SE = 0.09, *Z* =  − 2.93, *P* = 0.003; Table 1). Standard deviation among prairies was 0.0003.

**Table 1:** Best-fit generalized linear model representing variation in RIFA counts. We present results from the best-fit generalized linear model that examined the effect of RIFA suppression/treatment (i.e., prairies that were treated with insecticide and left untreated) and time on RIFA count data collected from April–July 2015 at our study site in the Attwater Prairie Chicken National Wildlife Refuge (APCNWR). This top-ranked model specified a Type 2 Negative Binomial error distribution with with intercepts randomly varying among prairies. A significantly higher rate of decrease or increased suppression of RIFA in treated areas over time was observed.

Variable	Estimate	SE	*Z*	*P* value
Intercept	2.319	0.332	6.99	<0.001
Treatment	−1.159	0.556	−2.08	0.037
Week	−0.091	0.040	−2.28	0.023
TreatmentTRT:Week	−0.257	0.088	−2.93	0.003^a^

**Notes.**

a Estimate = slope parameter or *β*; SE, standard error of the estimate; *Z*, Wald *Z*-test statistic; *P*, probability value.

Counts of native ants across 11 genera as well as counts of native ants whose taxonomic identities could not be ascertained were used. Mean number of native ants per sample were nearly 5 times greater in untreated sites (30.80 ± 6.10) compared to treated areas (6.28 ± 0.96; Fig. 3). A majority of observed native ants were cone ants (*Dorymyrmex* sp.), crazy ants (*Nylanderia* sp.), and *Forelius* sp. ants at treated (93.8%) and untreated (92.2%) sites. We failed to achieve convergence with the model that specified random variation in intercepts and slopes among prairies. The top-ranked model fitted native ant counts to a type-two negative binomial distribution, with intercepts varying randomly among prairies (Table S2). The top-ranked model solely specified a treatment effect with counts of native ants significantly lower in treated sites (*β* = −1.552, SE = 0.25, *Z* =  − 6.11, *P* <0.001; Table 2). Standard deviation among prairies was 0.158.

**Table 2:** Best-fit generalized linear model representing variation in Native Ant counts. We present results from the best-fit generalized linear mixed-effects model that examined the effect of RIFA suppression/treatment (i.e., prairies that were treated with insecticide and left untreated) and time on native ant count data collected from April–July 2015 at our study site in the Attwater Prairie Chicken National Wildlife Refuge (APCNWR). This top-ranked model specified a Type 2 Negative Binomial error distribution and random variation in intercepts among prairies. Native ant counts were significantly lower in treated areas.

Variable	Estimate	SE	*Z*	*P* value
Intercept	3.396	0.177	19.15	<0.001
TreatmentTRT	−1.552	0.254	−6.11	<0.001^a^

**Notes.**

a Estimate = slope parameter or *β*; SE, standard error of the estimate; *Z*, Wald *Z*-test statistic; *P*, probability value.

We observed nearly equal numbers of native non-ant invertebrates in treated (11.51 ± 1.46) and untreated sites (11.52 ± 1.23; Fig. 3). We observed beetles, crickets, juvenile grasshoppers, snails, spiders, and weevils among other native non-ant invertebrates. A majority of captures at both treated (97%) and untreated (95%) sites were small non-ant native invertebrates. Our top-ranked model fit these counts to a type two negative binomial distribution with intercepts randomly varying among prairies (Table S3). The best-fit model did not specify a treatment effect, instead specifying that counts of non-ant invertebrates decreased over time (*β* = −0.034, SE = 0.013, *Z* =  − 2.57, *P* = 0.01; Table 3).

**Table 3:** Best-fit generalized linear model representing variation in Non-Ant Invertebrate counts. We present results from the best-fit generalized linear mixed-effects model that examined the effect of RIFA suppression/treatment (i.e. prairies that were treated with insecticide and left untreated) and time on non-ant invertebrate count data collected from April–July 2015 at our study site in the Attwater Prairie Chicken National Wildlife Refuge (APCNWR). This top- ranked model specified a Type 2 Negative Binomial error distribution and random variation in intercepts among prairies. Non-ant invertebrate counts showed a significant decrease over time.

Variable	Estimate	SE	*Z*	*P* value
Intercept	2.664	0.114	23.33	<0.001
Week	−0.034	0.013	−2.57	0.01^a^

**Notes.**

a Estimate, slope parameter or *β*; SE, standard error of the estimate; *Z*, Wald *Z*-test statistic; *P*, probability value.

**Table 4:** List of competing models explaining variation in juvenile Houston Toad growth. Linear mixed-effects models were fit to repeated measures of Snout Urostyle Length of juvenile Houston Toads (*Bufo* [=*Anaxyrus*] *houstonensis*) maintained in terrestrial exclosures at Attwater Prairie Chicken National Wildlife Refuge. These data were collected from March–September, 2015. We examined the effect of RIFA suppression/treatment (i.e., prairies that were treated with insecticide and left untreated), initial/release density (four and six juvenile toads per exclosure), and time (i.e., week) on variation on SUL. We observed three models that had similar (Δ AIC_*c*_ ≤ 2) explanatory power with juvenile Houston Toad growth data. We report results from the model with the lowest AIC_*c*_ score which specified a higher order interaction of release density and time.

Model	*K*	AIC_*c*_	ΔAIC_*c*_
SUL∼Density*Week+(Week—Exclosure/IndID)	**11**	**5860.03**	**0.00**
SUL∼Week+(Week—Exclosure/IndID)	9	5,860.964	0.934
SUL∼Treatment+Density*Week+ (Week—Exclosure/IndID)	12	5,861.989	1.959^a^

**Notes.**

a *K*, Number of parameters; IndID, Individual ID of toad within an exclosure.

### Juvenile Houston toad growth and survivorship

**Figure 4:**
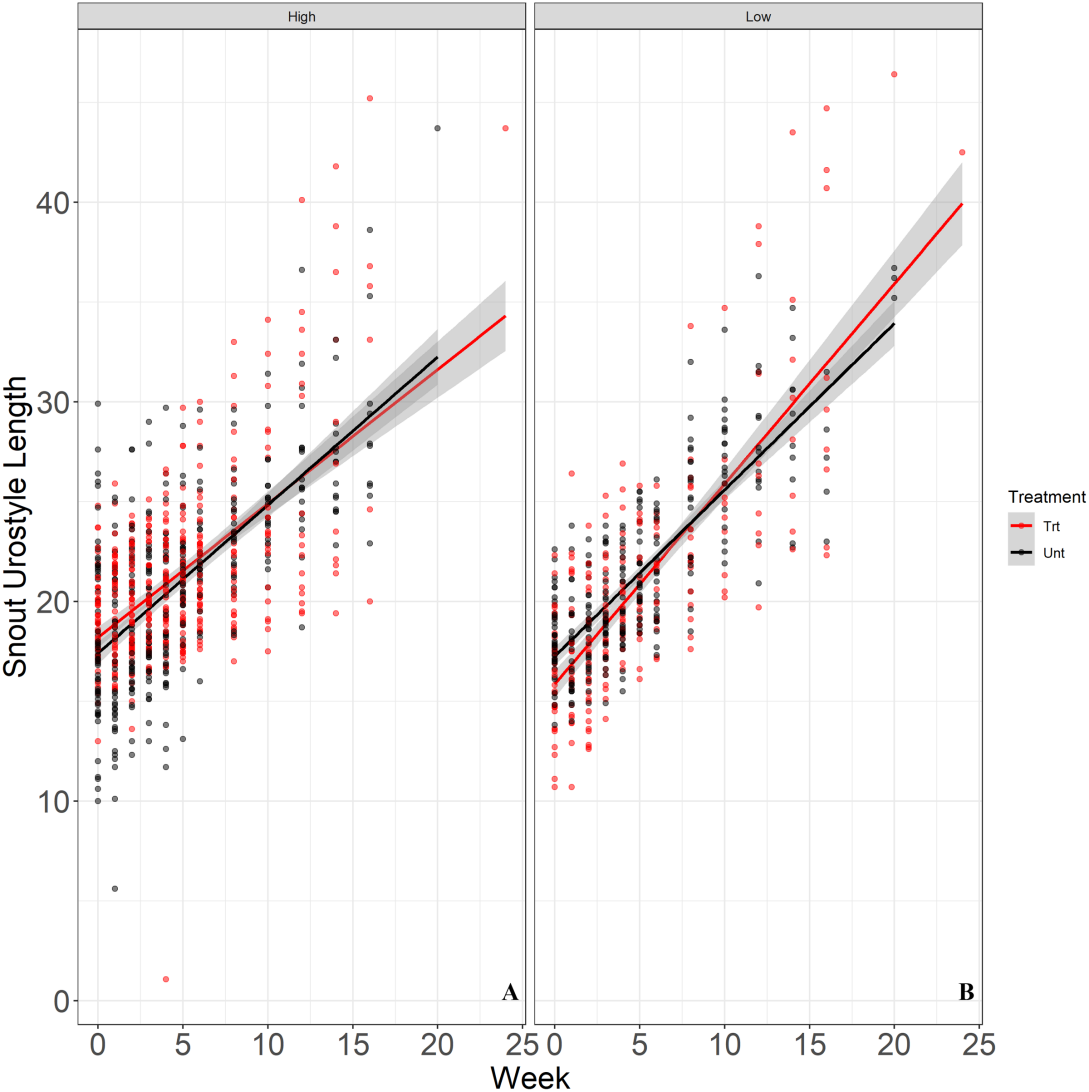
Rate of growth (*y*-axis) of juvenile Houston Toads maintained in high-density (six juveniles) and low-density (four juveniles) exclosures in RIFA treated (red) and untreated (black) sites at APCNWR. Our top-ranked linear mixed-effects model showed that Houston Toads (*Bufo* [=*Anaxyrus*] *houstonensis*) maintained in terrestrial exclosures with a low release density (B; four toads per exclosure) increased in SUL at a significantly greater rate than toads maintained in high release density exclosures (A; six toads per exclosure). The model did not include RIFA suppression/treatment as a predictor of variation in SUL of juvenile toads.

We released a total of 200 juvenile Houston Toads, with 100 juveniles each released in exclosures in sites treated and untreated for RIFA. The optimal random structure for our models allowed random variation in intercepts and slopes of exclosures and individual toads within exclosures, proving a better fit (ΔAIC_*c*_ = 68.36; Table S4) than the next best model which allowed random variation in slopes and intercepts of individual toads alone. In terms of fixed effects structure, we observed three competing models (ΔAIC_*c*_ ≤ 2; Table 4). The model with the lowest AIC_*c*_ score included a higher order interaction of release density with time, while the next model solely included time as a fixed factor (Table 4). We report from the top-ranked model that juvenile toads in exclosures with a lower release density showed a significantly greater rate of increase in SUL (*β* = 0.245, SE = 0.110, *t*_34.5_ = 2.226, *P* = 0.033; Table 5; Fig. 4). We saw more variation among individual toads within exclosures (SD = 2.09) and among exclosures (SD = 2.15) relative to the residual standard deviation (SD = 1.6).

**Table 5:** Top-ranked linear mixed-effects model explaining variation in juvenile Houston Toad growth. Linear mixed-effects models were fit to repeated measures of Snout Urostyle Length of juvenile Houston Toads (*Bufo* [=*Anaxyrus*] *houstonensis*) maintained in terrestrial exclosures at Attwater Prairie Chicken National Wildlife Refuge. These data were collected from March–September, 2015. We examined the effect of RIFA suppression/treatment (i.e., prairies that were treated with insecticide and left untreated), initial/release density (four and six juvenile toads per exclosure), and time (i.e., week) on variation on SUL. Our top-ranked model specified random variation in slopes and intercepts among exclosures and individual toads within exclosures. Further, juvenile toads in exclosures with low release density (four toads per exclosure) showed a significantly higher rate of increase in SUL relative to toads in high release density exclosures (six toads per exclosure).

Variable	Estimate	SE	Df	*t* value	*P* value
Intercept	17.847	0.526	35.65	33.91	<0.001
DensityLow	−1.191	0.760	38.70	−1.57	0.125
Week	0.532	0.076	30.78	7.00	<0.001
DensityLow:Week	0.245	0.110	34.49	2.23	0.033^a^

**Notes.**

Estimate, slope parameter or *β*; SE, standard error of the estimate; *Z*, Wald *Z*-test statistic; *P*, probability value

**Figure 5:**
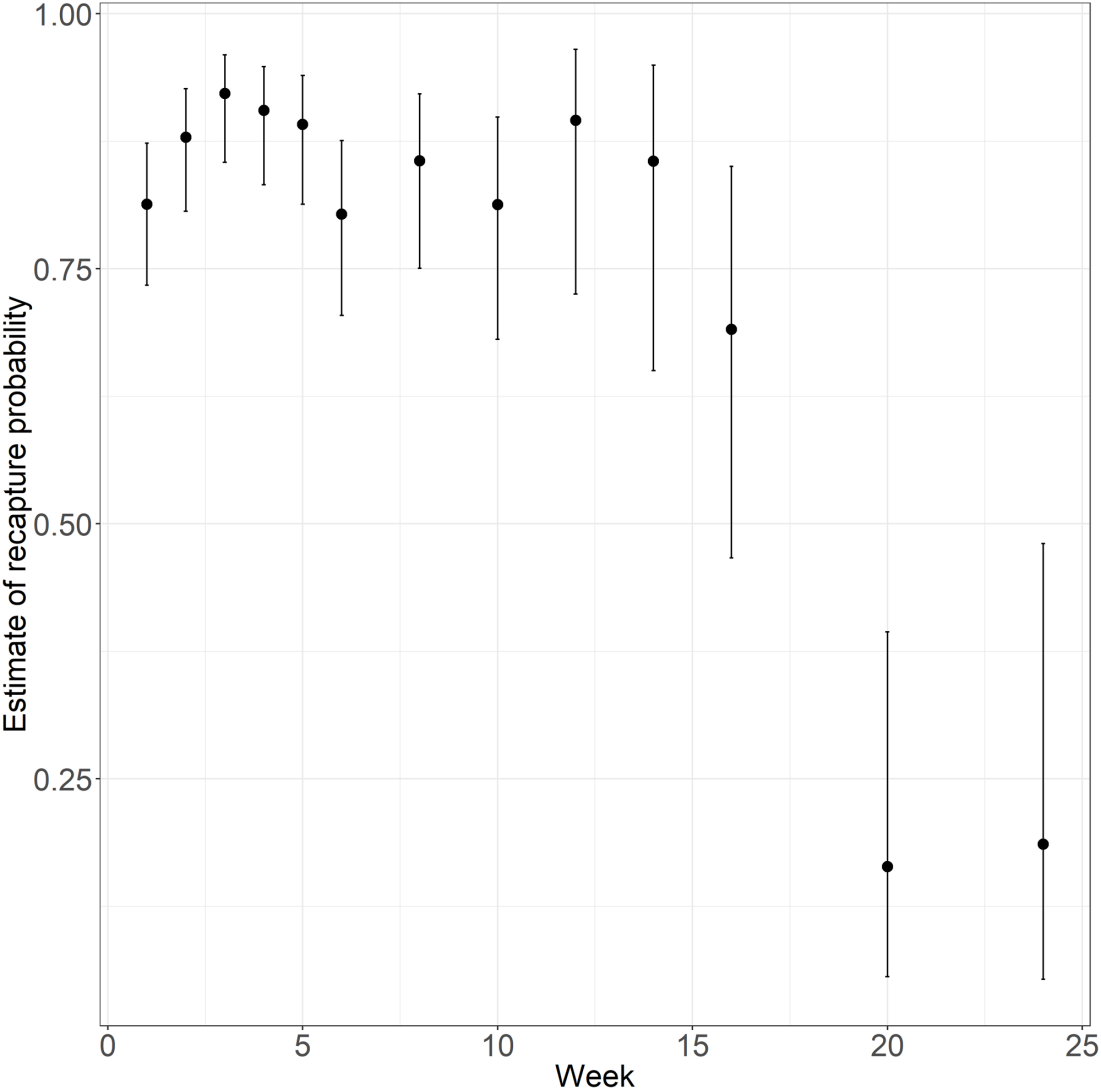
Temporal variation in recapture probability of juvenile Houston Toads (*Bufo* [=*Anaxyrus*] *houstonensis*) maintained in terrestrial exclosures at Attwater Prairie Chicken National Wildlife Refuge. Cormack-Jolly-Seber formulations were applied to live recapture data to estimate apparent survival (*φ*) and recapture probabilities (*p*) in two prairies each that were chemically treated and left untreated for Red Imported Fire Ants at APCNWR. The top-ranked model specified constant apparent survival and time-varying recapture probability, the latter being the lowest during the last two sampling occasions (i.e., week 20 and week 24).

The model that best fit our live-recapture data and received majority of support stipulated constant apparent survival and recapture probability that varied by time (*ϕ*_(.)_ *p*_(t)_; Table 6). The next best and competing model specified time-varying apparent survival and treatment-varying recapture probability (*ϕ*_(t)_ *p*_(g);_ΔQAIC _*c*_ = 0.33; Table 6). We report estimates from our top-ranked model. Apparent survival was estimated at 0.916 ± 0.008. We approximate overall apparent survival over the 24-week period to 0.12 (i.e., 0.916^24^). Recapture probabilities ranged from a minimum of 0.164 ± 0.084 to 0.922 ± 0.026 and were lowest over the last two sampling occasions (i.e., Week 20 and Week 24; Fig. 5).

## Discussion

RIFA have been considered a major threat to persistence of Houston Toad populations (IUCN/SSC Conservation Breeding Specialist Group, 1994). This was based off their potential to directly kill and eat Houston Toads as well as due to depletion of surface-active arthropods which constitute the prey base of Houston Toads (Bragg, 1960; Clarke, 1974; Potter Jr et al., 1984; Freed & Neitman, 1988; Porter & Savignano, 1990). However, Freed & Neitman’s (1988) report constitutes a single event of multiple juvenile toads being eaten by RIFA, while Brown, DeVolld & Forstner (2012) showed that RIFA activity around pond edges was highest at the time of Houston Toad metamorphs from these ponds but did not report on any incidents of RIFA predation on emergent toads. We conducted this experimental study at APCNWR as this site afforded us the opportunity to compare counts of RIFA, counts of native ants and non-ant invertebrates, and obtain repeated detections and measurements from juvenile Houston Toads that were maintained in experimental units in prairies that were chemically treated and left untreated for RIFA. We sought to determine the effect of chemical treatment on RIFA, native ant, and non-ant invertebrate abundance and determine if an expected decrease in RIFA in chemically treated sites facilitated a higher abundance of native ants and non-ant invertebrates as well as higher growth and survival rates in juvenile Houston Toads, relative to untreated sites. We observed that chemical treatment resulted in a discernible decrease in the number of RIFA. The insect growth regulator applied in treated sites resulted in an increased suppression of RIFA over time, as evidenced by a significant higher order interaction of time and treatment in our models (Calixto et al., 2007).

**Table 6:** List of Cormack–Jolly-Seber models that examine variation in juvenile Houston Toad survival. We report Cormack–Jolly–Seber models over a cumulative weight of ∼0.95. We evaluated the effect of RIFA suppression/treatment (i.e., prairies that were treated with insecticide and left untreated and time (i.e., week) on variation in survival of juvenile Houston Toads (*Bufo* [=*Anaxyrus*] *houstonensis*) maintained in terrestrial exclosures at Attwater Prairie Chicken National Wildlife Refuge. Live recapture data were collected from March–September, 2015. We assessed models using Quasi-likelihood Akaike Information Criterion scores that incorporated overdispersion and were corrected for a small sample size (ΔQAIC_*c*_). We determined the model that stipulated time-invariant apparent survival and time-varying recapture probability best fit our data and received majority of support.

Model	*K*	QAIC_*c*_	ΔQAIC_*c*_	*w*	∑*w*
*φ*_**(.)**_***p***_**(t)**_	**14**	**1333.85**	**0.00**	**0.35**	**0.35**
*φ*_(t)_*p*_(g)_	15	1334.20	0.35	0.29	0.64
*φ*_(t)_*p*_(.)_	14	1335.62	1.78	0.14	0.78
*φ*_(g)_*p*_(t)_	15	1335.73	1.88	0.14	0.92
*φ*_(g.t)_*p*_(.)_	27	1336.93	3.09	0.07	0.99

However, we also observed a significantly lower number of non-target native ants in RIFA-suppressed (i.e., treated) prairies. We did not observe a disproportionate effect of Extinguish^^®^^ Plus on RIFA abundance alone nor did we observe the expected positive effect of RIFA suppression on native ant assemblages. These results contrast with previous studies of native ant assemblages relative to RIFA abundance (Porter & Savignano, 1990; Calixto et al., 2007; Epperson & Allen, 2010). Caldwell (2015) however reported that chemical treatment suppressed RIFA and facilitated the recovery of native ants at APCNWR. It is important to note that Caldwell’s (2015) study included loamy and claypan prairie sites while all our prairies were coarse sand range sites and there may have been underlying differences in composition of native ant communities among our sites and Caldwell’s (2015) treated sites.

In fact, a majority of the native ant taxa that we captured at treated and untreated sites possibly included species such as cone ants (*Dorymyrmex* sp.), crazy ants (*Nylanderia* sp.), and *Forelius* sp. ants, which are known to coexist and exert a negative influence on RIFA survival (Porter & Savignano, 1990; Rao & Vinson, 2004; Calixto et al., 2007). We lack species-level resolution in our taxonomic identifications to be able to solidify this speculation. Our results on a higher number of native ants in untreated sites seem concordant to Morrow et al. (2015) who observed a substantially higher invertebrate count (i.e., inclusive of native ants) in 2012 at reference/untreated rather than treatment sites in APCNWR. The refuge is actively managed (i.e., patch-burning, removal of grazing, and disking fire breaks) to increase the abundance of early successional forbs and this is likely to maintain a relatively high baseline invertebrate abundance (Morrow et al., 2015). In addition, APCNWR received higher-than-average rainfall in the spring months of 2015 and it is possible that heavy rains and resultant abundant vegetation facilitated a higher insect abundance than could be influenced by RIFA predation (Caldwell, 2015).

We found an approximately equal number of non-ant invertebrates in untreated and treated prairies, which contrasts with a 75% reduction in non-ant arthropods in the presence of RIFA as reported by Porter & Savignano (1990) at the University of Texas at Austin’s Brackenridge Field Laboratory (BFL). However, our results do not contrast with a subsequent study that used comparable methods to sample ants and invertebrates at BFL (Morrison, 2002). The latter study reported a decrease in RIFA abundance and recovery of native ant and arthropod abundances in the 12 years following Porter & Savignano’s (1990) initial study. Morrison (2002) noted that the impacts of RIFA on native ant and arthropod assemblages may have been greatest during and shortly after the initial invasion. We speculate that our study may similarly report a ‘tempering in ferocity’ following the initial invasion at APCWNR. Additionally, our model reported a significant temporal decrease in counts of non-ant native invertebrates. We speculate that as we sampled from April through July, the decrease in non-ant invertebrates may be correlated with increasing summer temperatures.

Our models did not specify treatment as a predictor of variation in SUL of juvenile Houston Toads. Juvenile growth rates are therefore likely not affected by RIFA abundance at APCNWR. During the first season of his study, Caldwell (2015) reported much higher RIFA abundance from untreated sites at APCNWR compared to our results. We attribute these differences primarily to differences in the sizes of the sites sampled. The size of untreated sites in the second season of Caldwell’s (2015) study were similar to our study and so were the number of RIFA captured. However, we do note that the number of RIFA per sample even in untreated sites from our study are lower than abundances recorded in other studies with comparable methodology applied to similar (Apperson & Powell, 1984) and different (Landry, 2004) vegetation types. Therefore, while counts of RIFA were mathematically higher in untreated sites, RIFA abundance in untreated sites may not have been truly high enough to impact juvenile Houston Toads (Porter et al., 1991).

Analysis of live juvenile recaptures attempted to estimate apparent survival while accounting for detection rates <1.0. The model that best fit our recapture data showed that survival in juvenile Houston Toads did not differ among treated and untreated areas, meaning RIFA abundance at APCNWR probably did not affect juvenile survival. The top ranked model instead stipulated apparent survival that did not vary either by time or treatment. Recapture probabilities varied by time and we observed our lowest recapture rates during our last two sampling occasions. Additionally, the next best model with moderate support also specified no difference in survival among treated and untreated sites. Our findings appear to be concordant to a prior study that included APCNWR as one of the study sites, where variation in the probability of Attwater’s prairie-chicken brood survival to 2 weeks was largely explained by median invertebrate counts and invertebrate biomass (Morrow et al., 2015). Similar to our study, Morrow et al. (2015) did not observe differences in invertebrate counts or biomass among treated and untreated sites at APCNWR during the two-year study. Further, our study also conforms to Caldwell (2015) who also reported that chemical treatment to suppress RIFA did not lead to a higher northern bobwhite abundance. Additionally, juvenile Houston Toads did not differ in their growth rate among treated and untreated prairies. Since non-ant invertebrate abundance did not differ among our treatments, we could speculate that non-ant invertebrates may serve as potential food resources at low native ant abundances. Bragg (1960) reported that captive Houston Toads consumed various insects as well as a small Spadefoot Toad (*Scaphiopus* sp.) and Great Plains Toad (*Bufo* [=*Anaxyrus* ] *cognatus*). Additionally, an unpublished study that examined digestive tracts of 17 Houston Toads observed beetle remains in one individual and acrobat ants (*Crematogaster* sp.) in another (Potter Jr et al., 1984). Future *in-situ* studies that employ larger sample sizes are necessitated to confirm whether Houston Toads consume a relatively broad array of non-ant invertebrates as is indirectly indicated by our study.

With regards to our secondary goal, of evaluating whether juvenile Houston Toads can survive in coastal prairie habitat, we report that the overall survival of our toads following “emergence” to 24-weeks post emergence (0.12) appears higher than estimated by Greuter (2004), with the model estimating of the 200 juvenile toads initially released, 24 continued to survive at the end of 24 weeks. In fact, our estimate of apparent survival extrapolated to one year (0.01), appears comparable to the estimate required by Hatfield et al. (2004) in their Population Viability Analysis (PVA) for persistence of Houston Toad populations. Further, over the 24-week period, our juveniles showed a ∼1,500% increase in weight, i.e., growing from 0.77 ± 0.03 g to 12.33 ± 4.88 g and a ∼140% increase in SUL, i.e., growing from 18.05 ± 0.22 mm to 43.1 ± 0.6 mm. However, we do recognize that our method of artificial containment prevented the entry of vertebrate predators (e.g., snakes, birds), water tubs in exclosures mitigated chances of desiccation, and increased rainfall events in the spring (Caldwell, 2015) may possibly have inflated juvenile survival rates. Nonetheless, juvenile Houston Toads survived to the end of our study in coastal prairie habitat.

## Conclusions

We observed a decrease in RIFA in response to treatment with Extinguish^^®^^ Plus. However, juvenile Houston Toads did not demonstrate negative effects either in terms of their growth or survival from higher counts of Red Imported Fire Ants in untreated prairies. We believe our study is the first to mechanistically examine the influence of RIFA on survival and growth in juvenile Houston Toads in a recovering native prairie. As such, our findings regarding the potential lack of a relationship between RIFA abundance with growth and survival in juvenile Houston Toads are restricted to APCNWR. This refuge represents one of the largest, actively managed patches of coastal prairie (Morrow et al., 2015), and we recommend caution in extrapolating our observations to areas with management regimes that may be distinct from APCNWR. We emphasize the need for further studies that examine the impact of RIFA on Houston Toads at sites with different management regimes. Further our study demonstrates that the presence of RIFA at APCNWR may not hinder potential reintroduction efforts at APCNWR, either through direct predation of juvenile Houston Toads or by diminishing abundance of Houston Toad prey items. McHenry (2010) provided genetic evidence that adult males collected in 2007 from Colorado County were likely descendants from the Bastrop County source population released on APCNWR. Post-release monitoring of previous reintroduction efforts may have been hampered by budgetary constraints, which in turn precluded a thorough evaluation of the project (Quinn & Mays, 1987). Therefore, we believe prior notions on the unsuitability of APCNWR as a reintroduction site may need significant reevaluation.

We also observed significantly lower counts of native ants in treated prairies, meaning our bait-driven suppressant did not solely affect RIFA. Therefore, we draw attention to current strategies of using insecticidal baits to suppress RIFA numbers and that perhaps the species composition of native ant assemblages may be an important prior consideration before applying such strategies. As RIFA continues to be established in new areas (Ascunce et al., 2011), we emphasize the need for an increased number of experimental studies to assess the potential for non-target ant species impacts from chemical treatment. Additionally, restoring native invertebrate assemblages may also require reductions in the level of disturbance and restoration of undisturbed habitat (King & Tschinkel, 2006; Todd et al., 2008).

Further, we observed that juvenile Houston Toads survived to the end of our study and showed considerable growth. Our apparent survival estimates when extrapolated appear comparable to minimum juvenile survivorship required in simulations, for population persistence. We believe this finding highlights the enduring misconception that Houston Toad habitat consists primarily of canopied forest associated with sandy soils (Buzo, 2008; Forstner & Dixon, 2010). We believe our study demonstrates vegetation types such as open grasslands at APCNWR should be included in the regulatory definition of land cover types required to support Houston Toad populations, as either or both upland habitat and dispersal corridors. Our study necessitates further studies that compare survival rates for juvenile Houston Toads in non-forested and forested habitat.

Our findings demonstrate the utility of experimental studies/frameworks that examine the interactions between invasive species such as RIFA and threatened native species. A myriad of threats, that potentially include the spread of invasive species, are currently perceived as causes of decline in imperiled species. Wherever possible, experimental studies could be applied to potentially help rule out or confirm, at least in a site-specific manner, such perceived threats/cause of decline and enable the allocation of scarce resource towards focused recovery efforts for imperiled species.

##  Supplemental Information

10.7717/peerj.8480/supp-1 File S1 Pooled raw counts of Red Imported Fire Ants (*Solenopsis invicta*) from bait and pitfall traps that were set in treated and untreated sites at the Attwater Prairie Chicken National Wildlife Refuge (APCNWR) These raw data were used to compare RIFA counts among chemically treated and untreated sites.

10.7717/peerj.8480/supp-2 File S2 R code that examines variation in Red Imported Fire Ant (*Solenopsis invicta*) counts using generalized linear mixed effects models

10.7717/peerj.8480/supp-3 File S3 Pooled raw counts of native ants from bait and pitfall traps that were set in treated and untreated sites at the Attwater Prairie Chicken National Wildlife Refuge (APCNWR) These raw data were used to compare native ant counts among prairies that were chemically treated and left untreated for Red Imported Fire Ants (*Solenopsis invicta*).

10.7717/peerj.8480/supp-4 File S4 R code that examines variation in native ant counts using generalized linear mixed effects models

10.7717/peerj.8480/supp-5 File S5 Pooled raw counts of non-ant native invertebrates from bait and pitfall traps that were set in treated and untreated sites at the Attwater Prairie Chicken National Wildlife Refuge (APCNWR) These raw data were used to compare non-ant native invertebrate counts among prairies that were chemically treated and left untreated for Red Imported Fire Ants (*Solenopsis invicta*).

10.7717/peerj.8480/supp-6 File S6 R code that examines variation in non-ant native invertebrate counts using generalized linear mixed effects models

10.7717/peerj.8480/supp-7 File S7 Repeated raw measurements of snout urostyle length (SUL) and weight (g) of individually marked toads from outdoor mesocosms/exclosures at the Attwater Prairie Chicken National Wildlife Refuge These raw data were used to compare juvenile Houston Toad (*Bufo* [=*Anaxyrus* ] *houstonensis*) growth among prairies that were chemically treated and left untreated for Red Imported Fire Ants (*Solenopsis invicta*).

10.7717/peerj.8480/supp-8 File S8 R code to examine variation in growth of juvenile Houston Toads (*Bufo* [=*Anaxyrus* ] *houstonensis*) at the Attwater Prairie Chicken National Wildlife Refuge (APCNWR)

10.7717/peerj.8480/supp-9 File S9 Individual capture histories of juvenile Houston Toads (*Bufo* [=*Anaxyrus* ] *houstonensis*) that were maintained in exclosures at the Attwater Prairie Chicken National Wildlife Refuge (APCNWR) These raw data were used to compare apparent survival of juvenile toads among sites that were chemically treated and left untreated for Red Imported Fire Ants (*Solenopsis invicta*).

10.7717/peerj.8480/supp-10 File S10 R code to examine variation in apparent survival and recapture probability of juvenile Houston Toads (*Bufo* [=*Anaxyrus* ] *houstonensis*) at the Attwater Prairie Chicken National Wildlife Refuge (APCNWR)

10.7717/peerj.8480/supp-11 File S11 Raw counts of Red Imported Fire Ants (*Solenopsis invicta*), native ants, and non-ant native invertebrates at the Attwater Prairie Chicken National Wildlife Refuge (APCNWR) These raw data were used to visually examine invertebrate counts among sites that were chemically treated and left untreated for RIFA.

10.7717/peerj.8480/supp-12 File S12 Recapture probability estimates for juvenile Houston Toads (*Bufo* [=*Anaxyrus* ]* houstonensis)* generated from our best-fit model using the RMark interface These estimates were used to visually examine variation in recapture probability among sampling occasions.

10.7717/peerj.8480/supp-13 Table S1 Comparison of candidate models to examine variation in RIFA counts 12 candidate models were compared to examine the effect of RIFA suppression/treatment (i.e., prairies that were treated with insecticide and left untreated) and time on RIFA count data collected from April–July 2015 at our study site in the Attwater Prairie Chicken National Wildlife Refuge (APCNWR). We assessed models using Akaike Information Criterion scores corrected for a small sample size (AIC_*c*_). We determined models with random variation in intercepts were preferred. Subsequently, we determined the Type 2 Negative Binomial error distribution was preferred. Finally, a higher order interaction of treatment and time was the optimal fixed effects structure.

10.7717/peerj.8480/supp-14 Table S2 Comparison of candidate models to examine variation in Native Ant counts 12 candidate models were compared to examine the effect of RIFA suppression/treatment (i.e. prairies that were treated with insecticide and left untreated) and time on Native Ant count data collected from April–July 2015 at our study site in the Attwater Prairie Chicken National Wildlife Refuge (APCNWR). We assessed models using Akaike Information Criterion scores corrected for a small sample size (AIC_*c*_). We determined models with intercepts randomly varying among prairies were preferred. The model specifying random variation in intercepts and slopes failed to converge. Subsequently, we determined the Type 2 Negative Binomial error distribution was preferred. Finally, variation in native ant counts was best explained by the fixed factor of treatment.

10.7717/peerj.8480/supp-15 Table S3 Comparison of candidate models to examine variation in Non-Ant Invertebrate counts 12 candidate models were compared to examine the effect of RIFA suppression/treatment (i.e. prairies that were treated with insecticide and left untreated) and time on Non-Ant invertebrate count data collected from April–July 2015 at our study site in the Attwater Prairie Chicken National Wildlife Refuge (APCNWR). We assessed models using Akaike Information Criterion scores corrected for a small sample size (AIC_*c*_). We determined models with intercepts randomly varying among prairies were preferred. Subsequently, we determined the Type 2 Negative Binomial error distribution was preferred. Finally, variation in non-ant invertebrate counts was best explained by the fixed factor of time (i.e. week).

10.7717/peerj.8480/supp-16 Table S5 Comparison of candidate models to examine variation in juvenile Houston Toad growth 24 candidate models were compared to determine the effect of RIFA suppression/treatment (i.e. prairies that were treated with insecticide and left untreated), initial/release density (4 and 6 juvenile toads per exclosure), and time (i.e. week) on variation in Snout Urostyle Length of juvenile Houston Toads (*Bufo* [=*Anaxyrus* ] *houstonensis*) maintained in terrestrial exclosures at Attwater Prairie Chicken National Wildlife Refuge. Repeated measures of SUL were collected from March-August, 2015. We assessed models using Akaike Information Criterion scores corrected for a small sample size (AIC _*c*_). We determined models with slopes and intercepts randomly varying among exclosures and individual toads within exclosures were preferred. Subsequently, we observed the model specifying a higher order interaction of release density and time best explained variation in SUL of juvenile toads.

10.7717/peerj.8480/supp-17 Table S5 Comparison of candidate models to examine variation in juvenile Houston Toad survival 14 candidate models were compared to determine the effect of RIFA suppression/treatment (i.e. prairies that were treated with insecticide and left untreated and time (i.e. week) on variation in survival of juvenile Houston Toads (*Bufo* [=*Anaxyrus* ] *houstonensis*) maintained in terrestrial exclosures at Attwater Prairie Chicken National Wildlife Refuge. Live recapture data were collected from March–August, 2015 and analyzed using Cormack-Jolly-Seber models. We assessed models using Quasi-likelihood Akaike Information Criterion scores that incorporated overdispersion and were corrected for a small sample size (QAIC_*c*_). We determined the model that stipulated time-invariant apparent survival and time-varying recapture probability best fit our data and received majority of support.

10.7717/peerj.8480/supp-18 Figure S1 Current and historic range of Houston Toads (*Bufo* [=*Anaxyrus* ] *houstonensis*) in Texas Houston Toads have a restricted range with a currently known distribution in nine Texas counties (in blue) and extirpation of populations from three other counties (in tan). We conducted an experimental study at the Attwater Prairie Chicken National Wildlife Refuge in Colorado County to determine the impact of Red Imported Fire Ants (RIFA) on juvenile toad growth and survival. Map Data ©2019 Texas Department of Transportation.

## Additional Information and Declarations

### Field Study Permissions

The following information was supplied relating to field study approvals (i.e., approving body and any reference numbers): Individual marking and release of toads was authorized by a Federal Fish and Wildlife Permit TE039544-0 and a Texas Parks and Wildlife Department Scientific Research Permit SPR-0102-191.

### Data Availability

The following information was supplied regarding data availability: Raw data and code are available in the Supplementary Files.
