# Supplementary material for: Evaluating the effects of red imported fire ants (Solenopsis invicta) on juvenile Houston Toads (Bufo [=Anaxyrus] houstonensis) in Colorado County, TX
Source: PeerJ. 2020 Feb 10;8:e8480. doi: 10.7717/peerj.8480 (PMC7017801; doi:10.7717/peerj.8480)
Supplement: Table S1 — 12 candidate models were compared to examine the effect of RIFA suppression/treatment (i.e., prairies that were treated with insecticide and left untreated) and time on RIFA count data collected from April–July 2015 at our study site in the Attwater Prairie Chicken National Wildlife Refuge (APCNWR). We assessed models using Akaike Information Criterion scores corrected for a small sample size (AICc). We determined models with random variation in intercepts were preferred. Subsequently, we determined the Type 2 Negative Binomial error distribution was preferred. Finally, a higher order interaction of treatment and time was the optimal fixed effects structure. [file peerj-08-8480-s013.docx]

| Model | *K* | AIC*_c_* | ΔAIC*_c_* | Error Distribution |
| --- | --- | --- | --- | --- |
| Models varying in Random Structure | | | | |
| **RIFA~Treatment*Week + (1\|Prairie)** | **5** | **1198.90** | **0.00** | **Poisson** |
| RIFA~Treatment*Week + (Week\|Prairie) | 6 | 1199.53 | 0.64 | Poisson |
| Models varying in Error Distribution | | | | |
| **RIFA~Treatment*Week** + (1\|Prairie) | **5** | **665.84** | **0.00** | **NB2** |
| RIFA~Treatment*Week + (1\|Prairie) | 6 | 667.99 | 2.15 | ZI-NB2 |
| RIFA~Treatment*Week + (1\|Prairie) | 5 | 672.96 | 7.13 | NB1 |
| RIFA~Treatment*Week + (1\|Prairie) | 6 | 674.94 | 9.11 | ZI-NB1 |
| RIFA~Treatment*Week + (1\|Prairie) | 5 | 905.62 | 239.78 | ZI-Poisson |
| RIFA~Treatment*Week + (1\|Prairie) | 4 | 1198.90 | 533.10 | Poisson |
| Models varying in Fixed Factors |  |  |  |  |
| **RIFA~Treatment*Week** + (1\|Prairie) | **5** | **665.84** | **0.00** | **NB2** |
| RIFA~ Treatment+Week + (1\|Prairie) | 4 | 673.88 | 8.04 | NB2 |
| RIFA~Treatment + (1\|Prairie) | 3 | 684.32 | 18.48 | NB2 |
| RIFA~Week + (1\|Prairie) | 3 | 693.80 | 28.00 | NB2^[[1]](#footnote-1)^ |

| \|  \| \| --- \| \|  \| \|  \| \|  \| |
| --- | --- | --- | --- | --- |
|  |
|  |
|  |

1. *K*= Number of parameters; NB1 = Type 1 Negative Binomial; NB2 = Type 2 Negative Binomial; ZI-Poisson = Zero Inflated Poisson; ZI-NB1 = Zero-Inflated Type 1 Negative Binomial; ZI-NB2 = Zero-Inflated Type 2 Negative Binomial [↑](#footnote-ref-1)
