# Supplementary material for: Evaluating the effects of red imported fire ants (Solenopsis invicta) on juvenile Houston Toads (Bufo [=Anaxyrus] houstonensis) in Colorado County, TX
Source: PeerJ. 2020 Feb 10;8:e8480. doi: 10.7717/peerj.8480 (PMC7017801; doi:10.7717/peerj.8480)
Supplement: Table S2 — 12 candidate models were compared to examine the effect of RIFA suppression/treatment (i.e. prairies that were treated with insecticide and left untreated) and time on Native Ant count data collected from April–July 2015 at our study site in the Attwater Prairie Chicken National Wildlife Refuge (APCNWR). We assessed models using Akaike Information Criterion scores corrected for a small sample size (AICc). We determined models with intercepts randomly varying among prairies were preferred. The model specifying random variation in intercepts and slopes failed to converge. Subsequently, we determined the Type 2 Negative Binomial error distribution was preferred. Finally, variation in native ant counts was best explained by the fixed factor of treatment. [file peerj-08-8480-s014.docx]

| Model | *K* | AIC*_c_* | ΔAIC*_c_* | Error Distribution |
| --- | --- | --- | --- | --- |
| Models varying in Random Structure | | | | |
| **Native Ant~Treatment*Week + (1\|Prairie)** | **5** | **3019.22** | **0.00** | **Poisson** |
| Models varying in Error Distribution | | | | |
| **Native Ant~Treatment*Week +(1\|Prairie)** | **6** | **1411.16** | **0.00** | **NB2** |
| Native Ant~Treatment*Week+(1\|Prairie) | 7 | 1413.36 | 2.20 | ZI-NB2 |
| Native Ant~Treatment*Week+(1\|Prairie) | 7 | 1428.17 | 17.01 | ZI-NB1 |
| Native Ant~Treatment*Week+(1\|Prairie) | 6 | 1436.55 | 25.40 | NB1 |
| Native Ant~Treatment*Week+(1\|Prairie) | 6 | 2433.65 | 1022.5 | ZI-Poisson |
| Native Ant~Treatment*Week+(1\|Prairie) | 5 | 3019.22 | 1608.1 | Poisson |
| Models varying in Fixed Factors |  |  |  |  |
| **Native Ant~Treatment+(1\|Prairie)** | **4** | **1407.35** | **0.00** | **NB2** |
| Native Ant~Treatment+Week+(1\|Prairie) | 5 | 1409.33 | 1.98 | NB2 |
| Native Ant~Treatment*Week+(1\|Prairie) | 6 | 1411.16 | 3.81 | NB2 |
| Native Ant~Week+(1\|Prairie) | 4 | 1416.40 | 9.04 | NB2^[[1]](#footnote-1)^ |

| \|  \| \| --- \| \|  \| \|  \| \|  \| |
| --- | --- | --- | --- | --- |
|  |
|  |
|  |
|  |
|  |

1. *K*= Number of parameters; NB1 = Type 1 Negative Binomial; NB2 = Type 2 Negative Binomial; ZI-Poisson = Zero Inflated Poisson; ZI-NB1 = Zero-Inflated Type 1 Negative Binomial; ZI-NB2 = Zero-Inflated Type 2 Negative Binomial [↑](#footnote-ref-1)
