# Supplementary material for: Evaluating the effects of red imported fire ants (Solenopsis invicta) on juvenile Houston Toads (Bufo [=Anaxyrus] houstonensis) in Colorado County, TX
Source: PeerJ. 2020 Feb 10;8:e8480. doi: 10.7717/peerj.8480 (PMC7017801; doi:10.7717/peerj.8480)
Supplement: Table S5 — 24 candidate models were compared to determine the effect of RIFA suppression/treatment (i.e. prairies that were treated with insecticide and left untreated), initial/release density (4 and 6 juvenile toads per exclosure), and time (i.e. week) on variation in Snout Urostyle Length of juvenile Houston Toads (Bufo [=Anaxyrus ] houstonensis) maintained in terrestrial exclosures at Attwater Prairie Chicken National Wildlife Refuge. Repeated measures of SUL were collected from March-August, 2015. We assessed models using Akaike Information Criterion scores corrected for a small sample size (AIC c). We determined models with slopes and intercepts randomly varying among exclosures and individual toads within exclosures were preferred. Subsequently, we observed the model specifying a higher order interaction of release density and time best explained variation in SUL of juvenile toads. [file peerj-08-8480-s016.docx]

| Model | *K* | AIC*_c_* | ΔAIC*_c_* |
| --- | --- | --- | --- |
| Models varying in Random Factors | | | |
| **SUL~Treatment*Density*Week+**  **(Week\|Exclosure/IndID)** | **15** | **5870.58** | **0.00** |
| SUL~Treatment*Density*Week+  (Week\|Exclosure)+(Week\|IndID) | 15 | 5870.58 | 0.00 |
| SUL~Treatment*Density*Week+  (Week\|IndID) | 12 | 5938.94 | 68.36 |
| SUL~Treatment*Density*Week+  +(1\|Exclosure)+(1\|IndID) | 11 | 6537.68 | 667.1 |
| SUL~Treatment*Density*Week+  +(1\|Exclosure/IndID) | 11 | 6537.68 | 667.1 |
| SUL~Treatment*Density*Week+  +(1\|IndID) | 10 | 6554.35 | 683.76 |
| SUL~Treatment*Density*Week+  +(Week\|Exclosure) | 12 | 6640.79 | 770.21 |
| SUL~Treatment*Density*Week+  +(1\|Exclosure) | 10 | 6988.93 | 1118.3 |

| Model | *K* | AIC*_c_* | ΔAIC*_c_* |
| --- | --- | --- | --- |
| Models varying in Fixed Factors |  |  |  |
| **SUL~Density*Week+**  **(Week\|Exclosure/IndID)** | **11** | **5860.03** | **0.00** |
| SUL~ Week+  (Week\|Exclosure/IndID) | 9 | 5860.964 | 0.934 |
| SUL~Treatment+Density*Week+  (Week\|Exclosure/IndID) | 12 | 5861.989 | 1.959 |
| SUL~Treatment*Density+Week+  (Week\|Exclosure/IndID) | 12 | 5862.632 | 2.602 |
| SUL~Density+Week+  (Week\|Exclosure/IndID) | 10 | 5862.832 | 2.802 |
| SUL~Treatment+Week+  (Week\|Exclosure/IndID) | 10 | 5862.957 | 2.927 |
| SUL~Treatment*Density*Week+  (Week\|Exclosure/IndID) | 15 | 5863.575 | 3.545 |
| SUL~Treatment*Week+ Density*Week+  (Week\|Exclosure/IndID) | 13 | 5864.026 | 3.996 |
| SUL~Treatment+Density+Week+  (Week\|Exclosure/IndID) | 11 | 5864.821 | 4.791 |
| SUL~Treatment*Week+  (Week\|Exclosure/IndID) | 11 | 5864.961 | 4.931 |
| SUL~Treatment*Week+Density+  (Week\|Exclosure/IndID) | 12 | 5866.832 | 6.802 |
| SUL~Treatment*Density +  (Week\|Exclosure/IndID) | 11 | 5918.351 | 58.321 |
| SUL~Density+  (Week\|Exclosure/IndID) | 9 | 5918.46 | 58.43 |
| SUL~Treatment+  (Week\|Exclosure/IndID) | 9 | 5918.891 | 58.861 |
| SUL~Treatment+Density  (Week\|Exclosure/IndID) | 10 | 5920.428 | 60.398^[[1]](#footnote-1)^ |

1. *K* = Number of parameters; IndID = Individual ID of toad within an exclosure [↑](#footnote-ref-1)
