# Supplementary material for: Evaluating the effects of red imported fire ants (Solenopsis invicta) on juvenile Houston Toads (Bufo [=Anaxyrus] houstonensis) in Colorado County, TX
Source: PeerJ. 2020 Feb 10;8:e8480. doi: 10.7717/peerj.8480 (PMC7017801; doi:10.7717/peerj.8480)
Supplement: Table S5 — 14 candidate models were compared to determine the effect of RIFA suppression/treatment (i.e. prairies that were treated with insecticide and left untreated and time (i.e. week) on variation in survival of juvenile Houston Toads (Bufo [=Anaxyrus ] houstonensis) maintained in terrestrial exclosures at Attwater Prairie Chicken National Wildlife Refuge. Live recapture data were collected from March–August, 2015 and analyzed using Cormack-Jolly-Seber models. We assessed models using Quasi-likelihood Akaike Information Criterion scores that incorporated overdispersion and were corrected for a small sample size (QAICc). We determined the model that stipulated time-invariant apparent survival and time-varying recapture probability best fit our data and received majority of support. [file peerj-08-8480-s017.docx]

| Model | *K* | QAIC*_c_* | ΔQAIC*_c_* | *w* | ∑ *w* |
| --- | --- | --- | --- | --- | --- |
| **φ_(.)_*p*_(t)_** | **14** | **1333.85** | **0.00** | **0.35** | **0.35** |
| φ_(t)_*p*_(g)_ | 15 | 1334.20 | 0.35 | 0.29 | 0.64 |
| φ_(t)_*p*_(.)_ | 14 | 1335.62 | 1.78 | 0.14 | 0.78 |
| φ_(g)_*p*_(t)_ | 15 | 1335.73 | 1.88 | 0.14 | 0.92 |
| φ_(g.t)_*p*_(.)_ | 27 | 1336.93 | 3.09 | 0.07 | 0.99 |
| φ_(t)_*p*_(t)_ | 26 | 1341.38 | 7.54 | 0.01 | 1.00 |
| φ_(g.t)_*p*_(t)_ | 39 | 1342.38 | 8.53 | 0.00 | 1.00 |
| φ_(.)_*p*_(g.t)_ | 27 | 1348.12 | 14.27 | 0.00 | 1.00 |
| φ_(.)_*p*_(g)_ | 3 | 1349.20 | 15.35 | 0.00 | 1.00 |
| φ_(.)_*p*_(.)_ | 2 | 1350.45 | 16.60 | 0.00 | 1.00 |
| φ_(g)_*p*_(g)_ | 4 | 1351.12 | 17.27 | 0.00 | 1.00 |
| φ_(g)_*p*_(.)_ | 3 | 1352.41 | 18.57 | 0.00 | 1.00 |
| φ_(t)_*p*_(g.t)_ | 39 | 1354.93 | 21.08 | 0.00 | 1.00 |
| φ_(g.t)_*p*_(g.t)_ | 52 | 1357.84 | 23.99 | 0.00 | 1.00^[[1]](#footnote-1)^ |

1. *K* = Number of parameters; *w* = Model weight; ∑ *w* = Cumulative model weight [↑](#footnote-ref-1)
