# Supplementary figures and images for: Evaluating the effects of red imported fire ants (Solenopsis invicta) on juvenile Houston Toads (Bufo [=Anaxyrus] houstonensis) in Colorado County, TX
Source: PeerJ. 2020 Feb 10;8:e8480. doi: 10.7717/peerj.8480 (PMC7017801; doi:10.7717/peerj.8480)

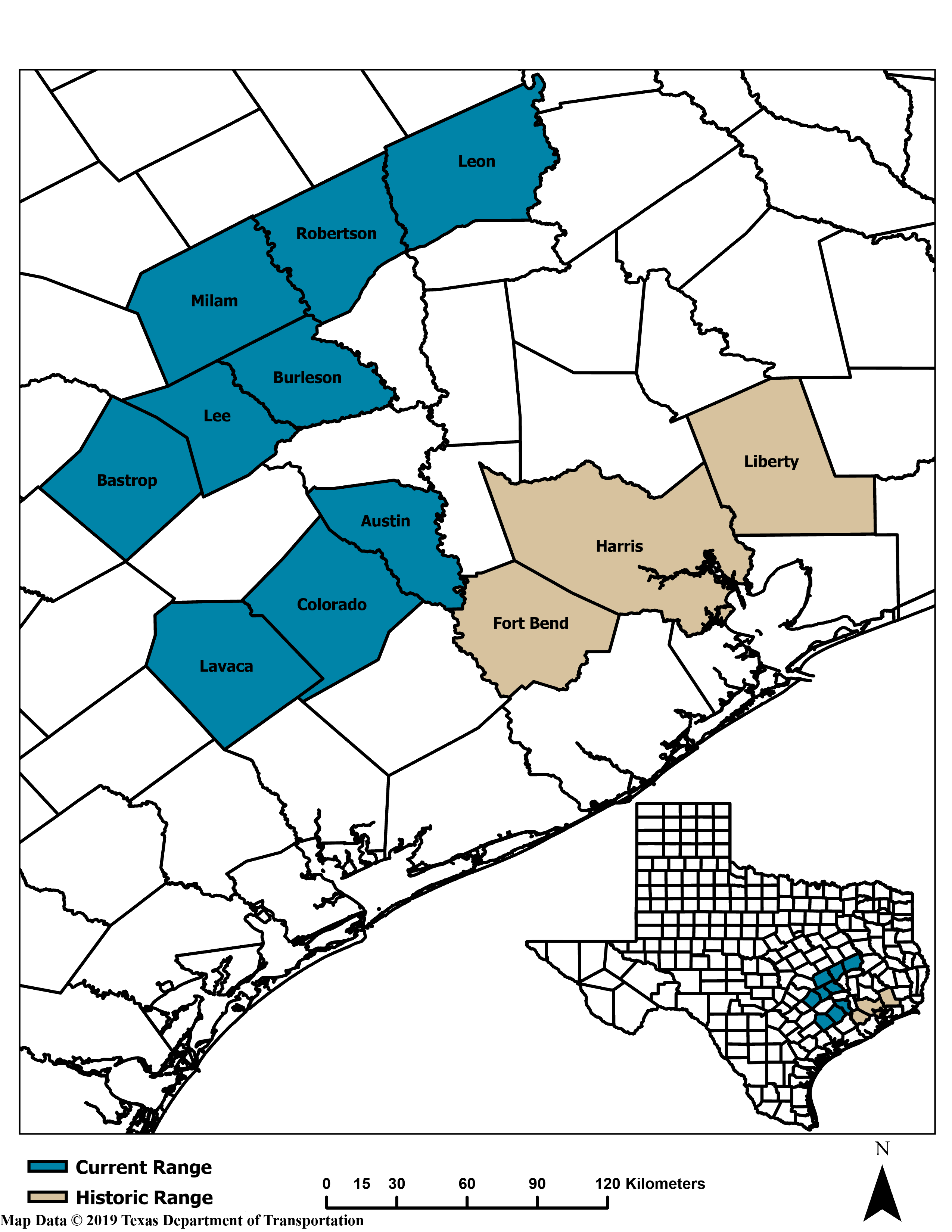

Supplement: Figure S1 — Houston Toads have a restricted range with a currently known distribution in nine Texas counties (in blue) and extirpation of populations from three other counties (in tan). We conducted an experimental study at the Attwater Prairie Chicken National Wildlife Refuge in Colorado County to determine the impact of Red Imported Fire Ants (RIFA) on juvenile toad growth and survival. Map Data ©2019 Texas Department of Transportation. [file peerj-08-8480-s018.png]
